# Differences between 9–11 year old British Pakistani and White British girls in physical activity and behavior during school recess

**DOI:** 10.1186/1471-2458-12-1087

**Published:** 2012-12-18

**Authors:** Tessa M Pollard, Yvonne C Hornby-Turner, Adarshini Ghurbhurrun, Nicola D Ridgers

**Affiliations:** 1 Physical Activity Laboratory, Department of Anthropology, Durham University, Dawson Building, South Road, Durham, DH1 3LE, UK; 2 Centre for Physical Activity and Nutrition Research, Deakin University, Burwood, Australia

**Keywords:** Physical activity, Recess, Playtime, South Asian, Pakistani, Ethnicity, Children, Girls, School, Accelerometry

## Abstract

**Background:**

School recess provides an important opportunity for children to engage in physical activity. Previous studies indicate that children and adults of South Asian origin are less active than other ethnic groups in the United Kingdom, but have not investigated whether activity differs within the shared school environment. The aim of this study was to test the hypothesis that British Pakistani girls aged 9–11 years are less active during recess than White British girls.

**Methods:**

In Study One, the proportion of recess spent by 137 White British (N = 70) and British Pakistani (N = 67) girls in sedentary behavior, moderate-to-vigorous activity (MVPA) and vigorous activity (VPA) was determined using accelerometry. In Study Two, 86 White British (N = 48) and British Pakistani (N = 38) girls were observed on the playground using the System for Observing Children’s Activity and Relationships during Play (SOCARP). Accelerometry data were collected during observations to allow identification of activities contributing to objectively measured physical activity.

**Results:**

Accelerometry data indicated that British Pakistani girls spent 2.2% (95% CI: 0.2, 4.3) less of their total recess time in MVPA and 1.3% (95% CI: 0.2, 2.4) less in VPA than White British girls. Direct observation showed that British Pakistani girls spent 12.0% (95% CI: 2.9, 21.1) less playground time being very active, and 12.3% (95% CI: 1.7, 23.0) less time playing games. Time spent being very active according to direct observation data correlated significantly with accelerometer-assessed time spent in MVPA and VPA, and time spent playing games correlated significantly with accelerometer-assessed time spent in VPA, suggesting that differences in behavior observed in Study Two may have contributed to the differences in time spent in MVPA and VPA in Study One.

**Conclusions:**

British Pakistani girls were less active than White British girls during school recess. Recess has been identified as a potentially important target for the delivery of physical activity interventions; such interventions should consider ways in which the activity levels of British Pakistani girls could be increased.

## Background

Low levels of physical activity have been reported in adults and children of South Asian origin living in the UK, Canada and the US [1-8] and are thought to contribute to the high risk of type 2 diabetes and cardiovascular disease in this group [9-11]. Activity levels are particularly low in people with origins in Bangladesh or Pakistan, and in women and girls [2,3,5]. This has been ascribed partly to concerns about modesty for Muslim women and girls, limiting time spent outdoors and in public places, as well as participation in mixed gender activities [12,13].

For children, physical activity during school recess makes an important contribution to activity, contributing up to 40% of daily recommended levels [14,15]. In the UK recess is mandatory and children are typically required to spend this time outside on the school playground, unless weather conditions make being outside impractical. One study of self-reported behavior in the playground in 11–15 year olds found no difference in activity between South Asian and White European children [16]. However, no study has used objective measures to compare activity levels in South Asian and White European children during recess. The use of objective methods is important not only because of concerns about the ability of children to recall often unstructured and varied playground activities [17], but also because any ethnic differences in views on the acceptability of active behaviors may lead to different biases in reporting in the two groups.

The aim of this study was to test the hypothesis that British Pakistani girls would be less active than White British girls during primary school recess. We based this hypothesis on the fact that activity appears to be even more constrained in Muslim girls, such as British Pakistani girls, than in other South Asian children, and posited that for this group constraints on activity may extend even into the shared environment of the playground. We compared activity levels and behaviors in British Pakistani girls and White British girls aged between 9 and 11 years. Rather than rely on self-report, we used physical activity monitors (accelerometry) and direct observation to assess activity. In Study One, the proportion of recess spent engaged in sedentary behavior, in moderate-to-vigorous physical activity (MVPA) and in vigorous physical activity (VPA) was determined using accelerometry. In Study Two, direct observation was used to assess time spent in different activities during recess, in combination with accelerometry to identify which observed activities contributed to objectively measured physical activity.

## Study one: methods

### Participants

One hundred and sixty-six White British and British Pakistani girls aged 9–11 years were recruited from years 5 and 6 of 7 primary (equivalent to US elementary) schools in a large conurbation in North East England. In three schools the researchers only approached year 5 girls, because head teachers were concerned about the impact of participation in the study on upcoming public examinations for year 6 girls. Informed written parental consent and verbal child assent to participate in this study, which was part of a larger study of activity and diet, were provided by all participants. Data were collected between January and December 2010. The research design and protocol received ethical approval from Durham University Ethics Committee.

### Instruments

#### Accelerometry

All girls were asked to wear the ActiGraph GT3X accelerometer on their right hip (ActiGraph, FL, USA) for two school days (as part of the larger study, which involved data collection for two school days and two weekend days). Data were recorded in 5 second epochs. For the purposes of this study data collected during all daily recess time, which consisted of a morning break (15 minutes at all schools) and a lunchtime break (lasting between 40 and 60 minutes depending on the school and including both recess and time to eat lunch), are reported. Data reduction was carried out using Actlife 5 software (version 5.8.1). Non-wear time was identified as periods of 60 minutes of consecutive zero counts to allow exclusion of girls who did not wear the monitor during recess. Time spent sedentary and in moderate activity and vigorous activity during recess on each day was extracted. We used the same cut-points as Owen et al. [5] in their study with this age group (<100 counts per minute (CPM) for sedentary activity, 2000–4000 CPM for moderate activity and >4000 CPM for vigorous activity). These cut-points are very close to those recently validated for children by Evenson et al. [18]. Time in MVPA was calculated by summing time in moderate and vigorous activity. For consistency with previous recess studies [19] the percentage of recess spent in each category was then calculated.

#### Questionnaire and interview

Parents were asked to record their child’s ethnicity, their own places of birth and their child’s first language as part of a general socio-demographic questionnaire. As part of data collection on diet, which was done by repeated 24-hour recall, children were asked whether they ate lunch at school or at home.

#### Weather

Information on the weather on days that girls wore the accelerometers was obtained from a weather station that was situated between 2 and 5 miles from each school. Maximum daily temperature was recorded, because temperature can affect activity level during recess [20,21]. Data on whether rainfall had occurred during recess were also recorded, as school staff may keep children inside during rain.

### Data analysis

Girls who recorded complete accelerometer data for recess over two days, including those who went home for lunch, were included in analyses, resulting in a final sample size of 137 (70 White British girls and 67 British Pakistani girls). Reasons for missing data were either failure to wear the accelerometer for two complete school days (N = 26) or absence from school (N = 3).

Multilevel modelling, performed using MLwiN (version 2.23) [22], was used to test for ethnic differences in activity. A three-level model was used, with day as level one, girl as level two and school as level three. Two models were run. Model 1 included only ethnicity as a fixed effect, and Model 2 additionally included fixed effects of year group, location of school lunch, total length of recess (since eating lunch, which is a sedentary behavior, will take up a higher proportion of time in schools where recess is shorter), temperature, and rainfall (as a dichotomous variable). Sedentary time, MVPA, and VPA were dependent variables in three separate models. Regression coefficients were assessed for significance using the Wald statistic. Statistical significance was set at p < 0.05.

## Study one: results

White British and British Pakistani girls were of a similar age and similarly distributed across school year groups (Table 1). British Pakistani girls were significantly more likely to return home for lunch during the mid-day recess and had a significantly longer total recess than White British girls. The days on which British Pakistani girls wore the accelerometers were significantly warmer and (on day 2 only) significantly drier than the days on which the White British girls collected data. Of the British Pakistani girls, 9 were born to parents who were both born in the UK, 13 were born to parents who were both born in Pakistan, and 31 were born to families with one Pakistani-born parent and one UK-born parent (data missing for 14 girls). Most (47) of the British Pakistani girls spoke English as their first language and 18 spoke Punjabi or Urdu as their first language (no data were available for 2 girls).

**Table 1:** Descriptive data for study one

	**White British (N = 70)**	**British Pakistani (N = 67)**
Age (years)	9.7 ± 1.2	9.9 ± 0.7
School year group		
5	53	51
6	17	16
Location of lunch		
School	70	57***
Home	0	10
Total recess time per day (minutes)	65.8 ± 6.7	68.2 ± 6.6*
Temperature (°C)		
Day 1	11.8 ± 6.9	14.5 ± 6.6*
Day 2	12.0 ± 7.0	14.6 ± 7.4*
Rainfall		
Day 1		
Yes	6	8
No	64	59
Day 2		
Yes	30	12**
No	40	55

*p < 0.05; **p < 0.01; ***p < 0.001.

The results of multilevel modelling (Table 2) showed that British Pakistani girls spent a greater proportion of their recess sedentary, a difference that approached statistical significance (p = 0.06). British Pakistani girls spent a significantly smaller proportion of their time in MVPA or in VPA in both the unadjusted and adjusted models. In the adjusted models the difference was 2.2% or 1.5 minutes of daily recess for MVPA and 1.3% or 0.9 minutes of daily recess for VPA. There was no significant difference between girls who went home for lunch and those who stayed at school in the time spent at any of these levels of activity. Nevertheless, to check whether the ethnic difference was the same for girls who stayed at school for lunch, models were run again excluding the girls who went home for lunch. In these models there was again no significant difference between the ethnic groups in time spent sedentary (p = 0.06), but a significant difference in time spent in MVPA and VPA (p < 0.05).

**Table 2:** Percentage of time spent in physical activity categories during recess by ethnicity (Study One)

	**Mean percentage of time ± SD**		**Mean difference in percentage of time ± 95% CI**	
	**White British**	**British Pakistani**	**Model 1 Estimate†**	**Model 2 Estimate‡**
Sedentary (%)	54.5 ± 11.7	57.3 ± 10.0	3.08 (−0.86, 7.02)	3.59 (−0.15, 7.33)
MVPA (%)	15.4 ± 6.5	12.9 ± 5.7	−2.42 (−4.50, −0.34)*	−2.24 (−4.28, −0.20)*
Vigorous activity (%)	5.9 ± 3.4	4.5 ± 3.1	−1.38 (−2.48, −0.28)**	−1.27 (−2.39, −0.15)*

† 3 level model with ethnicity as only fixed factor; ‡ includes additional adjustment for year group, location of lunch, total length of recess, temperature and rainfall; *p < 0.05; **p < 0.01; ***p < 0.001.

## Study two: methods

### Participants

Girls from 4 of the schools that participated in Study One were recruited to a further observational study. Informed written parental consent and verbal child assent to participate were provided by all 86 participants, of whom 63 (76%) also participated in Study One. Data were collected between June 2010 and February 2011. The research design and protocol received ethical approval from Durham University Ethics Committee.

## Instruments

### Observations

The System for Observing Children’s Activity and Relationships During Play (SOCARP), as described and validated by Ridgers et al. [20], was used to provide information on proportion of time spent at different levels of activity (lying, sitting, standing, walking or very active), in different activity types (sport, active games, sedentary activities, and locomotion such as walking or jogging that was not part of a sport or a game), in different groups sizes (alone, in a small group of 2–4 people or in a larger group) and in different social behaviors (prosocial physical, prosocial nonphysical, physical conflict or verbal conflict). Following the standard SOCARP protocol [20], each girl was observed over a 10-minute period using a time sampling technique by which a 10-second observation interval is followed by a 10-second recording interval, giving 30 observations for each girl. The presence or absence of play equipment in the playground was noted.

All data were collected by one of the authors (AG), following training by the last author. Training included familiarization with the protocol and codes, memorizing codes and coding conventions, and practicing observations using video examples of recess with trainer feedback. An assessment of observer reliability was conducted by comparing the observer scores of the to those of the last author. Initial training took 8 hours to reach acceptable agreement. Inter-observer reliability criteria was set at >80% using interval-by-interval agreement for each category.

#### Accelerometry

Girls were also asked to wear an ActiGraph GT3X during the recess when they were observed. Data for the 10 minute period of observation were extracted as for Study One, using the same cutpoints to determine proportion of observed time spent in sedentary time, MVPA and VPA.

#### Questionnaire

As part of a general socio-demographic questionnaire parents were asked to record their child’s ethnicity, their own places of birth and their child’s first language.

#### Weather

Information on the weather on the days that girls were observed was obtained from a local weather station as for Study One.

### Data analysis

SOCARP data were available for all 86 girls and accelerometer data were available for 71 (83%) girls. MLwiN was used to perform analyses, but because data were collected from only 4 schools, limiting higher-level variation, a single-level model was fitted, with school included as 3 dummy variables [23]. Two models were run to test for ethnic differences in SOCARP variables. Model 1 included only ethnicity and school as fixed effects, and Model 2 additionally included fixed effects of year group, availability of equipment (because equipment has been shown to be associated with increased activity [20,21]) and temperature. Rainfall was not included in these models as all children were observed while outside in the playground. Finally, Pearson correlation coefficients were calculated to test for associations between the SOCARP variables and accelerometer measures of physical activity.

## Study two: results

White British and British Pakistani girls were of a similar age and similarly distributed across school year groups, but play equipment was available to more British Pakistani than White British girls and the days on which British Pakistani girls were observed were significantly warmer (Table 3). Of the British Pakistani girls, 4 were born to parents who were both born in the UK, 8 were born to parents who were both born in Pakistan and 15 were born to families with one Pakistani-born parent and one UK-born parent (data missing for 11 girls). Most (25) of the British Pakistani girls spoke English as their first language and 10 spoke Punjabi or Urdu as their first language (no data were available for 3 girls).

**Table 3:** Descriptive data for study two

	**White British (N = 48)**	**British Pakistani (N = 38)**
Age (years)	9.9 ± 0.7	9.8 ± 0.8
School year group		
5	33	24
6	15	14
Equipment in playground		
Yes	32	33*
No	16	5
Temperature (°C)	11.5 ± 7.8	18.1 ± 7.2***

*p < 0.05; **p < 0.01; ***p < 0.001.

With respect to activity level, girls spent the majority of their time in the playground either walking or being very active. British Pakistani girls spent a significantly smaller proportion of observed time being very active than did White British girls. There were no other significant differences in time spent at different activity levels (Table 4). The most common activity type was locomotion, followed by sedentary activities and there was no difference by ethnic group in the proportion of time spent in these activity types, or in time spent playing sport. The raw data showed that White British and British Pakistani girls both spent about 16% of their time playing playground games, but after adjustment for school, year group, presence of equipment and temperature, a significant difference in time spent in playground games became apparent, with British Pakistani girls spending a significantly lower proportion of their time playing games. All girls spent the majority of their time in small groups and there were no ethnic differences in time spent in different group sizes. There was no difference between the two groups in the frequency of prosocial behavior. Conflict was very rarely observed and as a consequence it was not possible to test for ethnic differences in conflict.

**Table 4:** Percentage of time spent in physical activity categories during recess by ethnicity (Study Two)

	**Mean percentage of time ± SD**		**Mean difference in percentage of time ± 95% CI**	
	**White British**	**British Pakistani**	**Model 1 estimate†**	**Model 2 estimate‡**
Activity				
*Lying*	0.14 ± 0.96	0	NAº	NA
*Sitting*	4.2 ± 12.3	11.2 ± 24.2	3.78 (−5.47, 13.03)	2.43 (−6.78, 11.64)
*Standing*	17.6 ± 14.4	19.4 ± 18.0	6.91 (−1.05, 14.87)	5.80 (−2.14, 13.74)
*Walking*	41.0 ± 14.3	44.9 ± 20.5	2.89 (−5.81, 11.59)	5.03 (−3.34, 13.40)
*Very active*	36.5 ± 19.2	24.5 ± 17.1	−11.95 (−20.91, −2.99)**	−11.99 (−21.06, −2.92)**
Activity Type				
*Sedentary activities*	22.1 ± 17.5	26.3 ± 23.8	8.70 (−1.61, 19.01)	6.55 (−3.54, 16.64)
*Locomotion*	54.0 ± 24.9	50.4 ± 27.5	1.53 (−11.35, 14.41)	4.46 (−8.04, 16.96)
*Playground games*	16.4 ± 23.4	16.1 ± 21.3	−9.60 (−20.69, 1.49)	−12.33 (−23.01, −1.65)*
*Sports*	7.0 ± 18.9	6.9 ± 23.0	0.83 (−9.99, 11.65)	0.74 (−10.04, 11.52)
Group Size				
*Alone*	15.9 ± 17.4	13.6 ± 20.0	−5.97 (−15.24, 3.30)	−6.28 (−15.67, 3.11)
*Small*	76.0 ± 22.8	75.2 ± 28.9	10.29 (−2.45, 23.03)	8.56 (−4.16, 21.28)
*Medium/Large*	7.9 ± 18.9	11.2 ± 26.6	−3.09 (−14.67, 8.49)	−1.38 (−12.92, 10.16)
Social Behavior				
*Prosocial physical*	11.2 ± 13.7	11.0 ± 14.1	−0.44 (−7.46, 6.58)	−0.59 (−7.85, 6.67)
*Prosocial verbal*	1.1 ± 2.3	2.8 ± 8.6	NA	NA
*Physical conflict*	0.2 ± 1.1	0	NA	NA
*Verbal conflict*	0	0	NA	NA

† ethnicity and school included as fixed factors; ‡ includes additional adjustment for year group, presence of equipment and temperature; *p < 0.05; **p < 0.01; ***p < 0.001.

^º^NA = Not Applicable (no test conducted because behavior too infrequently recorded).

There were significant correlations between SOCARP activity level and activity type data and time spent in sedentary activity, MVPA and VPA as assessed by accelerometer (Table 5). Notably, time observed being very active was significantly positively correlated with time in MVPA and in VPA (and negatively correlated with time spent sedentary). Time spent playing playground games was significantly positively correlated with time in VPA, with an only slightly less strong correlation with time spent in MVPA. There were no significant correlations between time spent in different group sizes or in either physical or verbal prosocial behavior and accelerometer-assessed activity level.

**Table 5:** Pearson correlations between SOCARP variables and level of activity as assessed by accelerometer (N = 71)

	**Sedentary activity (% by accelerometer)**	**MVPA (% by accelerometer)**	**Vigorous activity (% by accelerometer)**
Activity			
*Sitting*	.49***	−.35**	−.29*
*Standing*	.54***	−.39**	−.28*
*Walking*	−.28*	−.12	−.25*
*Very active*	−.59***	.67***	.66**
Activity Type			
*Sedentary activity*	.69***	−.55***	−.47***
*Locomotion*	−.19	−.05	−.03
*Playground games*	−.05	.21	.23*
*Sports*	−.30**	.31**	.20
Group Size			
*Alone*	.05	−.12	−.11
*Small group*	.13	−.06	.02
*Medium/large group*	−.20	.20	.11
Social Behavior			
*Prosocial physical*	−.18	.18	.08
*Prosocial verbal*	.01	−.14	−.16

*p < 0.05; **p < 0.01; ***p < 0.001.

## Discussion

We found that during recess as a whole (which includes time spent eating lunch) (Study One), British Pakistani girls spent a smaller proportion of time in MVPA and in VPA than White British girls, and that when observed in the playground (Study Two) British Pakistani girls spent a smaller proportion of their time being very active and playing playground games. Time being very active according to direct observation data correlated significantly with accelerometer-assessed time spent in MVPA and VPA, and time spent playing games correlated significantly with accelerometer-assessed time spent in VPA, suggesting that differences in behavior (as seen in Study Two) may have contributed to the differences in time spent in MVPA and VPA (Study One). Study Two did not provide any evidence for an ethnic difference in the proportion of recess spent in sedentary behavior, but in Study One British Pakistani girls spent more time sedentary and this difference approached statistical significance. These data show that previous findings of low activity levels in South Asian children extend, in the case of British Pakistani girls, into the school playground. While other studies have identified ethnic differences in activity during school recess in the United States [24] and New Zealand [25] this is the first study to report an ethnic difference in physical activity during school recess in the United Kingdom, and the first to report a difference between children of South Asian and White European origin.

In contrast to our findings, Khunti et al. [16] observed no difference between 11–15 year old South Asian and White European children (boys or girls) living in inner city Leicester in self-report of at least one active behavior at recess. It is possible that they did not observe a difference between the groups because of their use of a simple self-report measure of activity. It is also possible that any difference in activity levels between South Asian and White European girls declines with age, so that the activity levels during recess of both reach the low levels observed by Khunti et al. It is worth noting, however, that most South Asian children in Leicester are of Indian origin [26], and it is also possible that activity levels in girls of Indian and of Pakistani origin differ.

The fact that British Pakistani girls were more likely to go home for lunch than White British girls did not explain their lower levels of accelerometer-assessed activity and, by definition, could not account for lower levels of observed activity in the playground. Previous studies have shown that children of normal weight tend to engage in more MVPA than overweight or obese children in general [27], and in the playground [20]. We did not collect anthropometric data for the current study, but a previous study with children from some of the same schools found no difference in body mass index (BMI) between White British and British Pakistani children [28]. It therefore seems unlikely that the lower levels of activity observed in British Pakistani girls were caused by greater BMI, although a higher level of adiposity in British Pakistani girls, as indicated by greater subscapular and triceps skinfold thicknesses [28], may be a cause, or, perhaps more! plausibly, an outcome, of lower levels of physical activity.

It is possible that British Pakistani girls are less active and less likely to participate in games in the playground because of concerns about modesty in a public, mixed gender environment. As Shaw [29] notes, in Pakistan the principles of purdah require girls and boys to be segregated from puberty onwards, and izzat, the honour of a family, can depend to a large extent on the behavior of daughters. In her work in the UK, Shaw found that traditional ideas about gender and sexuality influence the behavior and attitudes of the majority of young British Pakistanis. A previous British study showed that, in this age group, Asian children with origins on the Indian subcontinent (who were mostly Muslim) were less likely to interact with opposite sex children in the playground than were white children [30]. Since boys have consistently been shown to be more active on the playground than girls and tend to dominate both playground space and equipment for playing sport [14,21,31], avoidance of play with boys in Muslim South Asian girls may lead to lower levels of activity. The extent to which these issues influence physical activity levels should be investigated further, as this would inform future efforts to promote physical activity in British Pakistani girls. Ultimately, studies should be conducted to examine the feasibility and effectiveness of interventions, perhaps including the provision of single sex play spaces, to increase physical activity levels in this group.

While British South Asian children, especially women and girls and those with origins in Pakistan and Bangladesh, are known to have low overall physical activity levels, this is the first study to investigate whether this difference holds within the school environment, when children of all ethnicities share the same physical environment. The fact that lower activity levels were observed using both accelerometry and direct observation adds weight to our results. However, we acknowledge that collection of accelerometry data over more than two school days would have been desirable. The fact that data collection took place on warmer and drier days for British Pakistani girls may have influenced our results, despite statistical adjustment for temperature and rainfall. Our sample size for Study Two was limited and larger studies may identify further differences in the playground behavior of White British and British Pakistani girls. Given differences in activity levels observed in adults it would be interesting to compare activity at recess in other South Asian groups, according to country of origin. There may also be differences depending on whether parents grew up in Pakistan or the UK [32], or according to the extent to which families or girls subscribe to concerns about modesty, but our sample size did not allow us to test for such effects. Finally, studies should be conducted to test whether differences such as that observed here are confined to girls, as seems possible, or are present in boys as well.

## Conclusions

Previous studies have identified low activity levels in children of South Asian origin living in the UK. In 9–11 year old British Pakistani girls, this finding extends into the school playground.

Given that school recess contributes significantly to children’s overall activity levels, it has been identified as a potentially important target for the delivery of physical activity interventions [21,33]. The needs of particular groups of children must be considered in the design of such interventions; the necessity for school environment interventions to consider the specific requirements of girls in general has previously been identified [34], but those of specific ethnic groups have not. One possibility in the case of Muslim girls of South Asian origin is that single-sex environments could be created to encourage active play in this group (for example in school halls). Future research could also compare recess interventions to interventions delivered as after-school activities.

## Competing interests

The authors declare that they have no competing interests.

## Authors’ contributions

TP conceived the study, conducted the analyses and wrote the manuscript. YHT, AG and NR contributed to the planning and design of the study and supplied comments on the manuscript. YHT and AG collected the data. All authors read and approved the final version of the manuscript.

## Pre-publication history

The pre-publication history for this paper can be accessed here:

http://www.biomedcentral.com/1471-2458/12/1087/prepub
